# Evaluating the significance of ECSCR in the diagnosis of ulcerative colitis and drug efficacy assessment

**DOI:** 10.3389/fimmu.2024.1426875

**Published:** 2024-08-07

**Authors:** Bin Feng, Yanqiu Zhang, Longwei Qiao, Qingqin Tang, Zheng Zhang, Sheng Zhang, Jun Qiu, Xianping Zhou, Chao Huang, Yuting Liang

**Affiliations:** ^1^ Center for Clinical Laboratory, The First Affiliated Hospital of Soochow University, Suzhou, Jiangsu, China; ^2^ Institute of Clinical Pharmacology, Anhui Medical University, Key Laboratory of Anti-inflammatory and Immune Medicine, Ministry of Education, Anhui Collaborative Innovation Center of Anti-inflammatory and Immune Medicine, Hefei, Anhui, China; ^3^ Center for Reproduction and Genetics, School of Gusu, The Affiliated Suzhou Hospital of Nanjing Medical University, Suzhou Municipal Hospital, Nanjing Medical University, Suzhou, Jiangsu, China; ^4^ State Key Laboratory of Genetic Engineering, School of Life Sciences, Fudan University, Shanghai, China; ^5^ Department of Laboratory, Bozhou Hospital Affiliated to Anhui Medical University, Bozhou, Anhui, China; ^6^ Department of Laboratory, Anhui Medical University, The First Affiliated Hospital of Anhui Medical University, Hefei, Anhui, China

**Keywords:** ulcerative colitis, ECSCR, machine learning, diagnosis, biomarker

## Abstract

**Background:**

The main challenge in diagnosing and treating ulcerative colitis (UC) has prompted this study to discover useful biomarkers and understand the underlying molecular mechanisms.

**Methods:**

In this study, transcriptomic data from intestinal mucosal biopsies underwent Robust Rank Aggregation (RRA) analysis to identify differential genes. These genes intersected with UC key genes from Weighted Gene Co-expression Network Analysis (WGCNA). Machine learning identified UC signature genes, aiding predictive model development. Validation involved external data for diagnostic, progression, and drug efficacy assessment, along with ELISA testing of clinical serum samples.

**Results:**

RRA integrative analysis identified 251 up-regulated and 211 down-regulated DEGs intersecting with key UC genes in WGCNA, yielding 212 key DEGs. Subsequently, five UC signature biomarkers were identified by machine learning based on the key DEGs—THY1, SLC6A14, ECSCR, FAP, and GPR109B. A logistic regression model incorporating these five genes was constructed. The AUC values for the model set and internal validation data were 0.995 and 0.959, respectively. Mechanistically, activation of the IL-17 signaling pathway, TNF signaling pathway, PI3K-Akt signaling pathway in UC was indicated by KEGG and GSVA analyses, which were positively correlated with the signature biomarkers. Additionally, the expression of the signature biomarkers was strongly correlated with various UC types and drug efficacy in different datasets. Notably, ECSCR was found to be upregulated in UC serum and exhibited a positive correlation with neutrophil levels in UC patients.

**Conclusions:**

THY1, SLC6A14, ECSCR, FAP, and GPR109B can serve as potential biomarkers of UC and are closely related to signaling pathways associated with UC progression. The discovery of these markers provides valuable information for understanding the molecular mechanisms of UC.

## Introduction

Ulcerative colitis (UC) is a chronic inflammatory bowel disease (IBD) that commonly affects individuals in their young and middle-aged years. Clinical symptoms encompass abdominal cramps, pus, mucus, and bloody diarrhea (1–3). The global incidence of UC ranges from 10.5 to 14 cases per 100,000 people annually, with a prevalence of approximately 246.7 cases per 100,000 individuals (4). Nevertheless, a consistent increase in UC incidence is observed in Western countries, coupled with significant surges in South America and East Asia. Furthermore, UC onset is occurring at younger ages, with both developed and developing countries experiencing a rise in UC cases among children (5). Given the challenges associated with curing UC, its propensity for recurrence, and the heightened risk of cancer (6), early initiation of treatment for remission and long-term maintenance to prevent recurrence constitute crucial strategies. A comprehensive understanding of the disease’s pathogenesis and the identification of new biomarkers may offer insights for early diagnosis and monitoring disease progression, ultimately contributing significantly to the improvement of overall health outcomes.

Endoscopy and tissue biopsy continue to serve as the exclusive methods for confirming a diagnosis of UC and evaluating disease activity and severity, albeit causing significant discomfort for patients with UC (7). Furthermore, the reluctance of patients to undergo this technique can lead to delayed diagnosis (8). The development of UC is due to a complex interaction between the host’s genetic background, microbial changes, and environmental factors, resulting in improper and chronic activation of the mucosal immune system (9–13). The identification of biomarkers could play a crucial role in facilitating precise diagnosis and selecting therapeutic approaches for UC patients. Combining C-reactive protein and fecal calreticulin levels may provide some benefits in the ongoing evaluation and monitoring of UC progression; however, their efficacy is limited (14). Therefore, the urgent need to identify distinctive markers of UC is paramount for facilitating early diagnosis, assessing disease progression, uncovering new pathogenic mechanisms, and enabling the prediction and development of therapeutic strategies for UC. Moreover, Aminosalicylates (5-ASA) and corticosteroids are fundamentally used in treating and controlling mild to moderate UC (15, 16). Immunomodulators and biologic therapies, such as anti-TNF-alpha agents (infliximab, adalimumab, golimumab), are effective in reducing UC inflammation and improving disease prognosis (11). With the increasing incidence of UC, the number of patients with refractory UC—who exhibit poor or no response to conventional medications, prolonged disease duration, and recurrent exacerbations—is also rising (17). Therefore, a thorough investigation of biomarkers associated with therapy response in UC patients is essential, along with the development of new approaches to enhance response rates.

**Figure 1:**
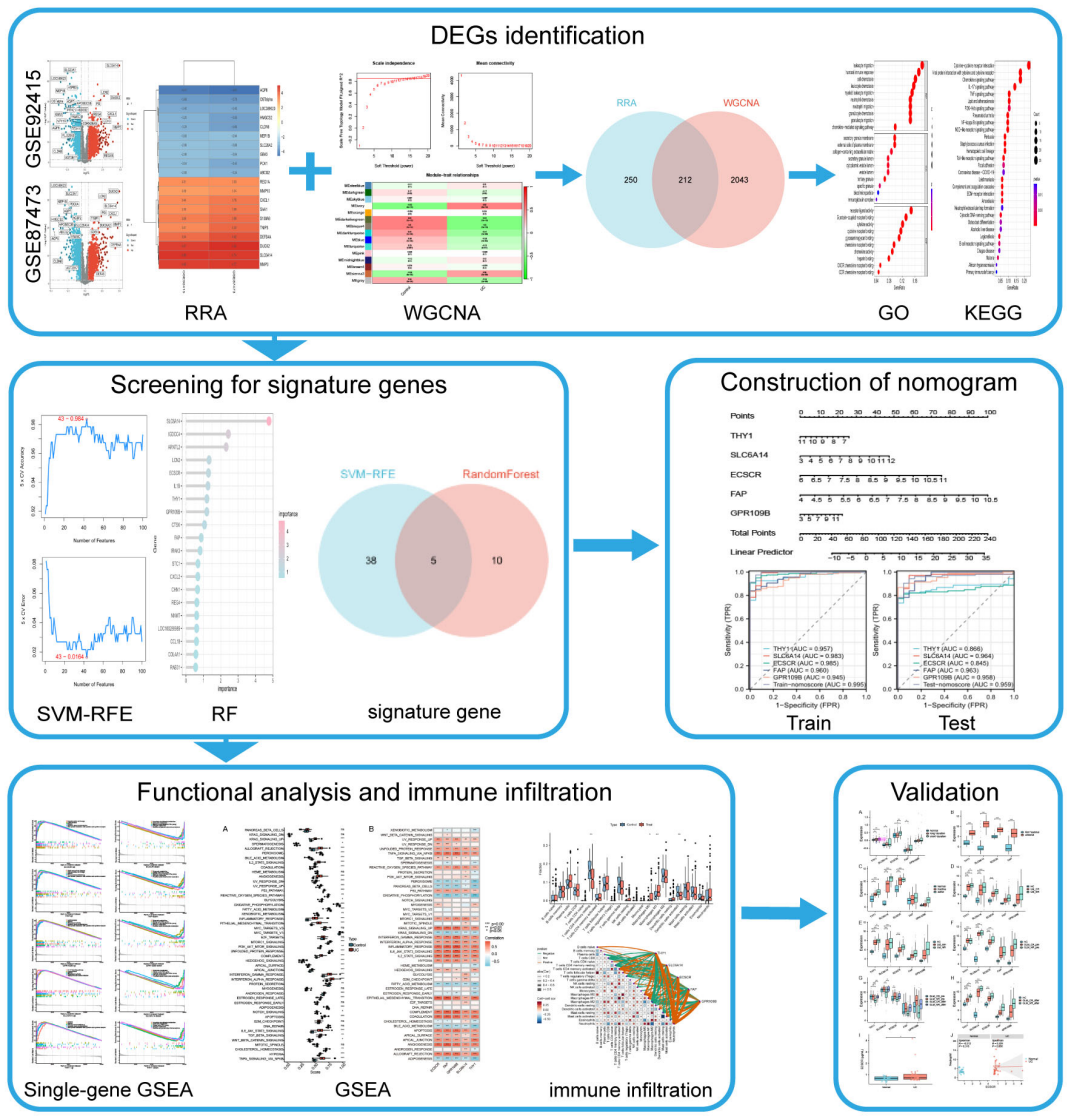
Flow diagram of the analysis process.

Gene chip assay technology, combined with second-generation sequencing technology and bioinformatics analysis, is currently widely employed for exploring the pathological characteristics of various diseases and identifying potential novel biomarkers, including UC (9, 10, 18). In this study, using transcriptomic data from a substantial number of intestinal mucosal biopsies, differential genes were identified through Robust Rank Aggregation (RRA) analysis and intersected with UC key genes identified by Weighted Gene Co-expression Network Analysis (WGCNA). Subsequently, machine learning algorithms were employed to identify UC signature genes, which were then utilized to develop predictive models. The diagnostic performance of both the modeled genes and models, as well as their relationship with disease progression and drug efficacy, were validated using external data. The diagnostic efficacy of the modeled genes for UC was further confirmed through ELISA testing of clinical serum samples. The objective of this work is to provide new insights into the early diagnosis of UC. The flow chart of this study is illustrated in **Figure 1**.

## Materials and methods

### Data collection

The gene expression datasets for UC were retrieved from the Gene Expression Omnibus (GEO) database (https://www.ncbi.nlm.nih.gov/). Six specific datasets, namely GSE92415, GSE87473, GSE11223, GSE107499, GSE53306, and GSE206285 (**Table 1**), were selected for inclusion in our study. Subsequently, both gene expression profiles and corresponding clinical information were downloaded for further analysis.

**Table 1:** Microarray information.

GEO	Platform	Tissue	Samples (number)			Attribute
			Total	UC	Control	
GSE92415	GPL13158	Colon	183	162	21	Training set
GSE87473	GPL13158	Colon	21	106	21	Validation set
GSE11223	GPL1708	Colon	202	129	73	Validation set
GSE107499	GPL15207	Colon	119	75	44	Validation set
GSE53306	GPL14951	Colon	40	28	12	Validation set
GSE206285	GPL13158	Colon	382	364	18	Validation set

### Identification of differentially expressed genes (DEGs)

The GSE92415 and GSE87473 datasets were selected for differential gene expression analysis. The R package “limma” was used to identify DEGs using |log2fold change (FC)| > 0.5 and adjusted P *<* 0.05 as the cutoff criteria (19–21). The outcomes were visualized using “ggplot2” R packages. Following this, both datasets were amalgamated and subjected to an analysis of common DEGs using the RRA method (22). The lists of up- and down-regulated genes in each dataset were sorted based on logFC. Subsequently, all gene lists were integrated utilizing the “RobustRankAggreg” package.

### Construction of the co-expression network by WGCNA

The R package “WGCNA” was used for gene co-expression network analysis. The WGCNA algorithm is a systems biology approach for characterizing patterns of correlation between genes in different samples (23). Initially, a hierarchical cluster analysis is performed to filter the discrete samples. Subsequently, the optimal soft threshold power (β) was chosen to calculated the adjacency matrix, which was then transformed into a topological overlap matrix (TOM). The identification of different modules was achieved by the dynamic tree-cutting method, specifically filtering modules containing more than 50 genes. Finally, Pearson correlation analysis was used to calculate the correlation between modules and traits. Modules with the most significant correlation coefficients were then selected for further analysis. Intersecting genes identified through both RRA and WGCNA methods were deemed key differential genes for UC.

### Functional enrichment analysis of key genes

The R package “org.Hs.eg.db” was used to convert the gene names of the DEGs into gene IDs. The R package “clusterProfiler” was implemented to conduct Gene Ontology (GO) and Kyoto Encyclopedia of Genes and Genomes (KEGG) functional enrichment analysis to assess gene-related biological processes (BP), molecular functions (MF), cellular components (CC), and signaling pathways (24, 25). The results were plotted using the R packages “enrichplot” and “ggplot2”.

### Screening signature genes by machine learning

To further screen key differential genes, this study employed two machine learning algorithms, support vector machine-recursive feature elimination (SVM-RFE) and Random Forest (RF). SVM-RFE was performed by “caret” package in R at 10-fold cross-validation to determine the variables at the max accuracy (26). RF algorithm was used to rank gene importance using the R package “randomForest” (27). Finally, the intersection of the two machine learning algorithms was identified as signature genes.

### Construction and validation of a diagnostic nomogram in UC

The signature genes were used to construct the nomogram in training set (GSE92415) by the R package “rms” (28). The nomoscore based on signature gene expression was calculated by linearly combining coefficients from logistic regression and expression levels. Subsequently, Receiver operating characteristic (ROC) curves were plotted via the R “pROC” package to assess the diagnostic ability of the nomogram model (29). In addition, the nomogram model was tested in the test set (GSE87473).


$$\text{Nomoscore}=\text{Coef }+\sum_{i=1}^{n}Expr_{i}\times Coef_{i}\;$$

Coef is regression coefficient; Coefi stands for the coefficient of genei; Expr_i_ is the expression of gene_i_.

### Gene set enrichment analysis

GSEA was performed to explore the possible function of signature genes using the hallmark gene sets (h.all.v7.5.1.symbols), which were obtained from the Molecular Signatures Database (https://www.gsea‐msigdb.org/gsea/msigdb/index.jsp) (30). R package “GSVA” was applied to compute a hallmark gene set score based on gene expression levels for each sample.

### Immune infiltration analysis

To analyze immune infiltration, the CIBERSORT algorithm assessed differential immune cell presence in the colon mucosa of both UC patients and healthy controls (31). Additionally, the Spearman’s correlation analysis was performed to investigate the relationship between immune cells and the identified signature genes (32).

### External validation of signature genes and correlation with drug efficacy

GSE11223, GSE107499, GSE53306, and GSE206285 datasets were downloaded for external validation. Differences in expression levels of signature genes in different types of UC and drug efficacy were validated using the Wilcoxon rank sum test, and the results were visualized using the boxplot.

### Enzyme-linked immunosorbent assay

Serum samples were collected from 40 UC patients and 25 normal controls from the First Affiliated Hospital of Soochow University. Optical density (OD) was measured at 450 nm using a microplate reader according to the manufacturer’s instructions, and the concentration of ECSCR in the samples was calculated from a standard curve. In this experiment, the detection limit for human ECSCR was 0.1-5μg/ml. The medical Ethics Committee of the First Affiliated Hospital of Soochow University approved this study.

### Statistical analysis

All statistical analysis was performed using R (version4.3.1) (https://www.r-project.org) and associated R packages. A significance level of P < 0.05 was used for all analyses to indicate statistical significance.

## Results

### Identification of key differential genes and functional enrichment analysis

**Figure 2:**
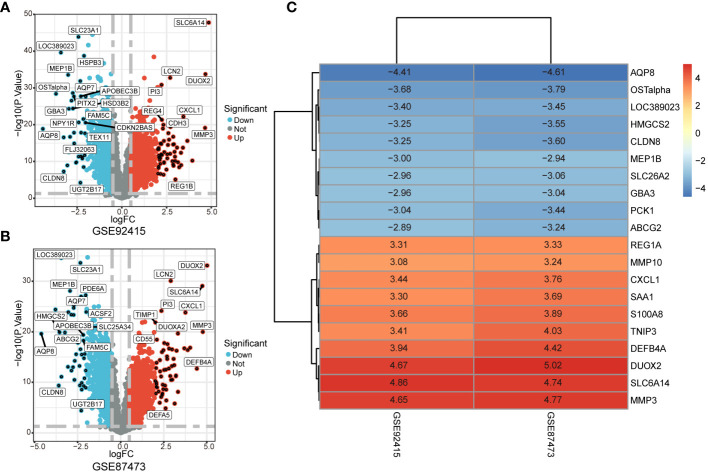
Identification of DEGs associated with UC. **(A, B)** Volcano plot shows DEGs in GSE92475 and GSE87473 datasets. **(C)** Heatmap of the top 10 up- and down-regulated DEGs identified in the RRA analysis.

The GSE92415 and GSE87473 datasets underwent separate differential analyses, leading to the identification of 2761 and 3096 DEGs based on the criteria of logFC > 0.5 and adjusted P < 0.05, respectively (**Figures 2A, B**). Following this, RRA was employed to integrate the results from the two cohort analyses, revealing 251 up-regulated and 211 down-regulated DEGs. The heatmap illustrates the degree of upregulation or downregulation observed in the top 10 DEGs across both datasets (**Figure 2C**).

**Figure 3:**
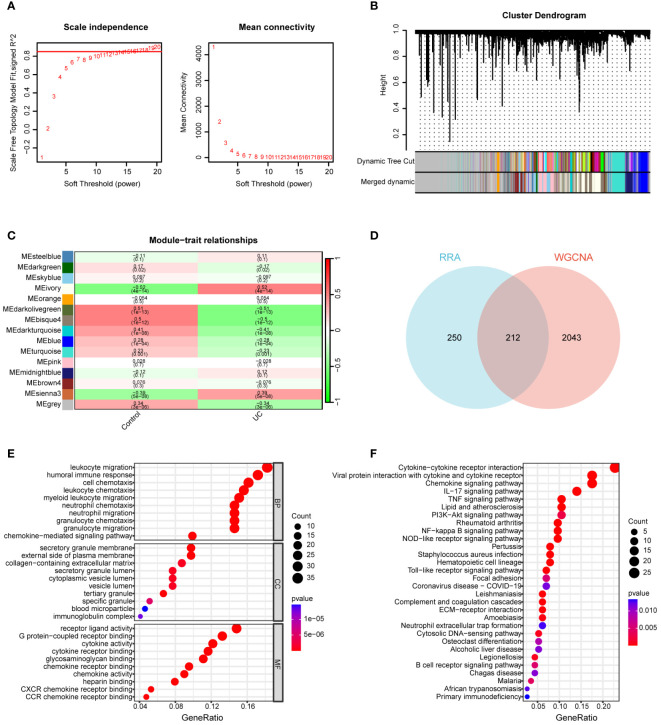
Construction of WGCNA network. **(A)** Soft threshold power screening and scale-free network construction. **(B)** Cluster dendrogram of the co-expression network modules (1-TOM). **(C)** Heatmap shows the correlation between module eigengenes and UC. **(D)** Venn diagram of 212 overlapping genes between ivory module genes and RRA. **(E)** GO enrichment analysis of 212 overlapping genes. **(F)** KEGG enrichment analysis of 212 overlapping genes.

The GSE92415 dataset, in conjunction with the WGCNA software package, was utilized to discern functional clusters linked to UC patients. In the construction of gene co-expression networks, a soft threshold of β=16 was selected, aligning with a correlation coefficient nearing 0.85 (**Figure 3A**). Post-merging comparable modules using a MEDissThres of 0.3, a total of 15 modules were generated (**Figure 3B**). An analysis of module-trait relationships through a heatmap unveiled that the ivory modules exhibited the most robust correlation with UC progression (r = 0.52, P = 4e-14) (**Figure 3C**).

A total of 212 genes were identified as differentially expressed in both the RRA and WGCNA analyses, designating them as key differential genes through the application of a Venn diagram (**Figure 3D**). To thoroughly elucidate the biological processes and pathways associated with these DEGs, we conducted GO and KEGG enrichment analyses. In the GO analysis, DEGs exhibited significant enrichment in the following processes: leukocyte migration, humoral immune response, cell chemotaxis, leukocyte chemotaxis, secretory granule membrane, external side of the plasma membrane, collagen-containing extracellular matrix, receptor ligand activity, G protein-coupled receptor binding, cytokine activity, and cytokine receptor binding (**Figure 3E**). For the KEGG analysis, genes displayed notable enrichment in cytokine-cytokine receptor interaction, chemokine signaling pathway, IL-17 signaling pathway, TNF signaling pathway, PI3K-Akt signaling pathway, and rheumatoid arthritis (RA) (**Figure 3F**).

### Using machine learning to identify the signature gene of UC

**Figure 4:**
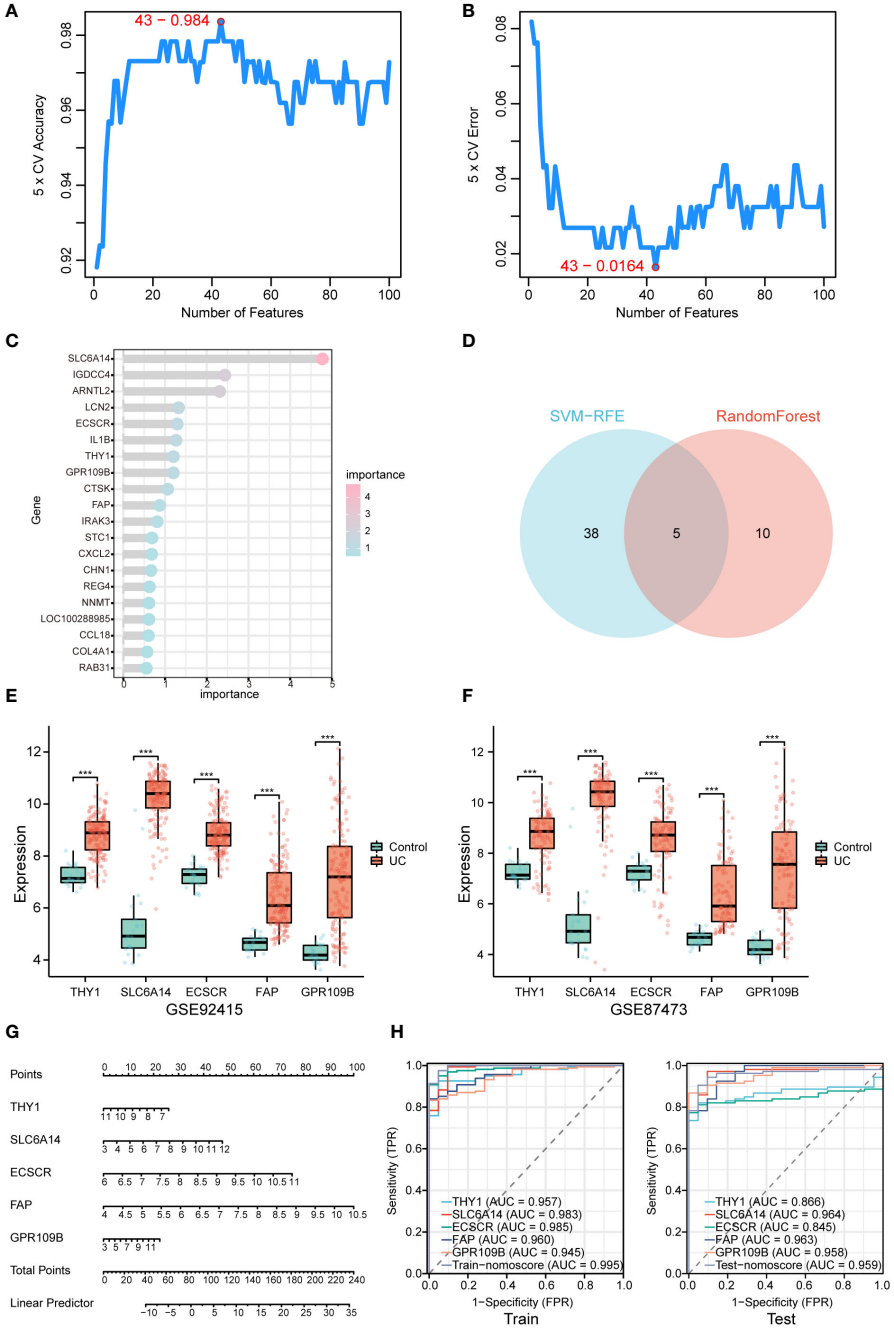
Screening for signature genes. **(A, B)** SVM-RFE algorithm was used to select the genes. **(C)** The genes were selected based on the RandomForest. **(D)** Venn diagram for two algorithmic. **(E, F)** Signature genes expression in GSE92415 dataset and GSE87473 dataset. **(G)** Nomogram for signature genes. **(H)** The ROC curve of the nomogram in the model set (GSE92415) and internal validation data (GSE87473). ***P < 0.001.

Two machine learning algorithms, SVM-RFE and RF, were employed to identify potential diagnostic biomarkers from DEGs. Utilizing the SVM-RFE algorithm, we pinpointed 43 genes with distinct features, achieved by minimizing cross-validation error (**Figures 4A, B**). Additionally, RF highlighted the top 15 genes (**Figure 4C**) and identified a total of 5 UC signature biomarkers—THY1, SLC6A14, ECSCR, FAP, and GPR109B—by taking the intersection of the genes (**Figure 4D**).

To enhance our comprehension of the predictive value associated with these signature genes for UC, an analysis was undertaken on the five signature biomarkers across two datasets (**Figures 4E, F**). The results unveiled a significant upregulation of expression in UC patients for these 5 characterized genes. Following this observation, a logistic regression model was constructed utilizing these 5 characterized genes (refer to **Figure 4G**). The Area Under the Curve (AUC) values were determined to be 0.995 and 0.959 in the model set and internal validation data, respectively. This signifies that the model exhibited superior efficacy in distinguishing between UC and healthy control samples compared to the individual prediction of the 5 characterized genes (as depicted in **Figure 4H**).

### Investigation of specific signaling mechanisms associated with the UC signature gene

**Figure 5:**
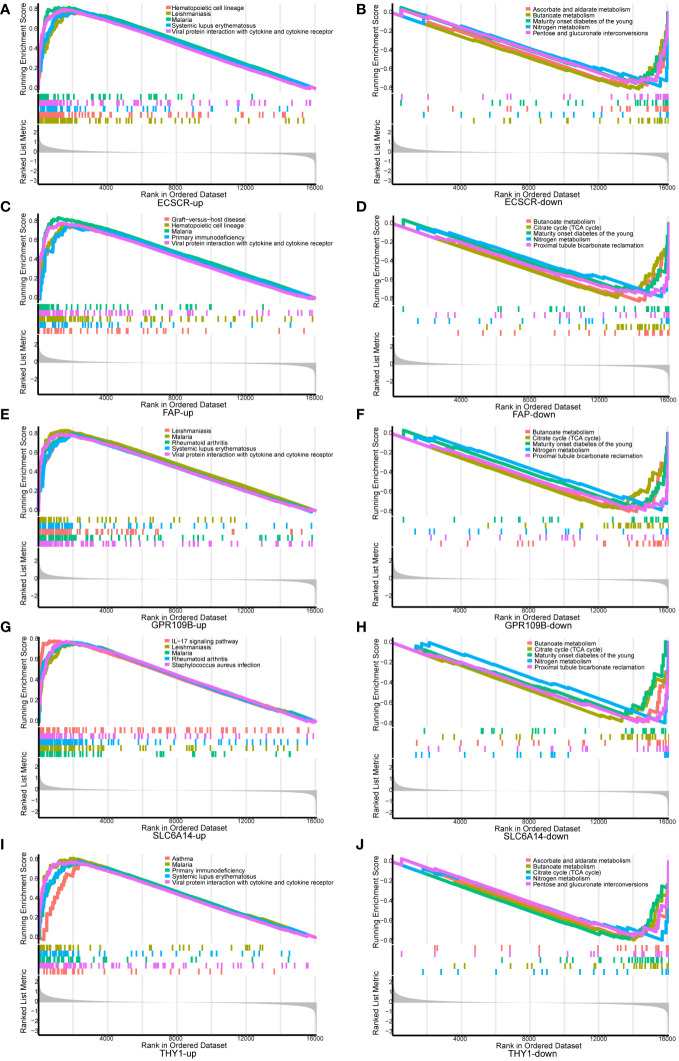
GSEA analysis of signature genes. **(A, B)** ECSCR, **(C, D)** FAP, **(E, F)** GPR109B, **(G, H)** SLC6A14, **(I, J)** THY1.

GSEA analysis was conducted to scrutinize the signaling pathways implicated in the five signature genes and explore their impact on signaling pathways related to UC progression (**Figure 5**). The results demonstrated that genes associated with elevated THY1 expression were notably enriched in primary immunodeficiency and viral protein interaction with cytokine and cytokine receptor. In contrast, genes linked to low THY1 expression showed significant enrichment in ascorbate and aldarate metabolism, butanoate metabolism and citrate cycle (TCA cycle). For genes associated with high expression of SLC6A14, there was significant enrichment in the IL-17 signaling pathway and RA. Conversely, genes related to low SLC6A14 expression displayed enrichment in butanoate metabolism and the citrate cycle (TCA cycle). Genes associated with high ECSCR expression were significantly enriched in hematopoietic cell lineage and viral protein interaction with cytokine and cytokine receptors, while genes linked to low ECSCR expression showed significant enrichment in ascorbate and aldarate metabolism, as well as butanoate metabolism. Similarly, genes associated with high FAP expression exhibited significant enrichment in Hematopoietic cell lineage and primary immunodeficiency. In contrast, genes associated with low FAP expression displayed significant enrichment in butanoate metabolism and the citrate cycle (TCA cycle). Lastly, genes associated with high GPR109B expression displayed significant enrichment in RA and viral protein interaction with cytokine and cytokine receptors. Conversely, genes associated with low GPR109B expression showed significant enrichment in butanoate metabolism and the citrate cycle (TCA cycle).

**Figure 6:**
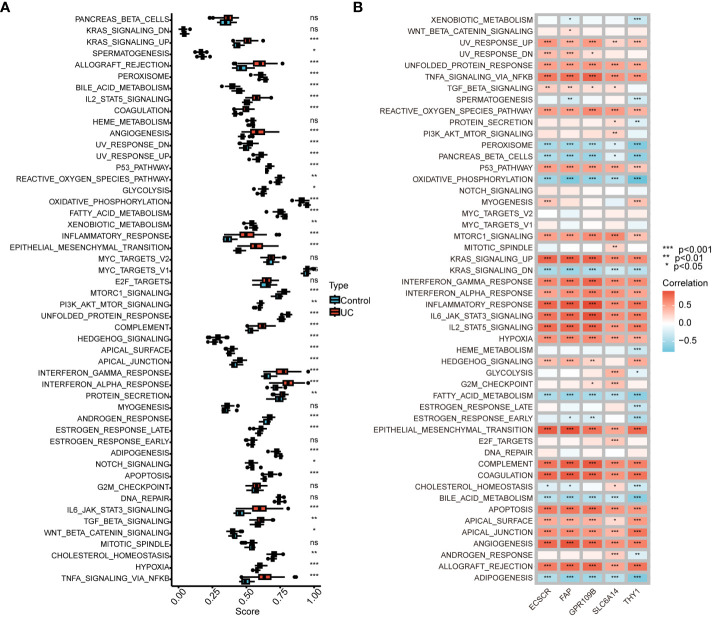
Correlation between UC signature genes and Hallmark pathway. **(A)** Comparison of GSVA scores in the Hallmark pathway between normal control and UC patients. **(B)** Correlation between signature genes and GSVA scores for the Hallmark pathway. ns, no significance. *P < 0.05, **P < 0.01, ***P < 0.001.

We conducted an analysis to compare GSVA scores in the Hallmark pathway between normal controls and UC patients. The results revealed a significant and predominant upregulation of the Hallmark pathway in UC patients. Specifically, upregulation was observed in KRAS signaling, IL2-STAT5 signaling, interferon gamma response, interferon alpha response, IL6-JAK-STAT3 signaling, and TNFA signaling via NFKB. Conversely, bile acid metabolism, oxidative phosphorylation, and fatty acid metabolism were significantly downregulated (**Figure 6A**). Furthermore, we explored the correlation between genes in our model and GSVA scores for the Hallmark pathway. The analysis demonstrated a positive correlation between gene expression in the model and the Hallmark pathway upregulated in UC patients, as well as a negative correlation with the Hallmark pathway downregulated in UC patients (**Figure 6B**). These findings provide additional support for the involvement of genes in our model in the progression of UC through various pathways.

### Immune infiltration and correlation

**Figure 7:**
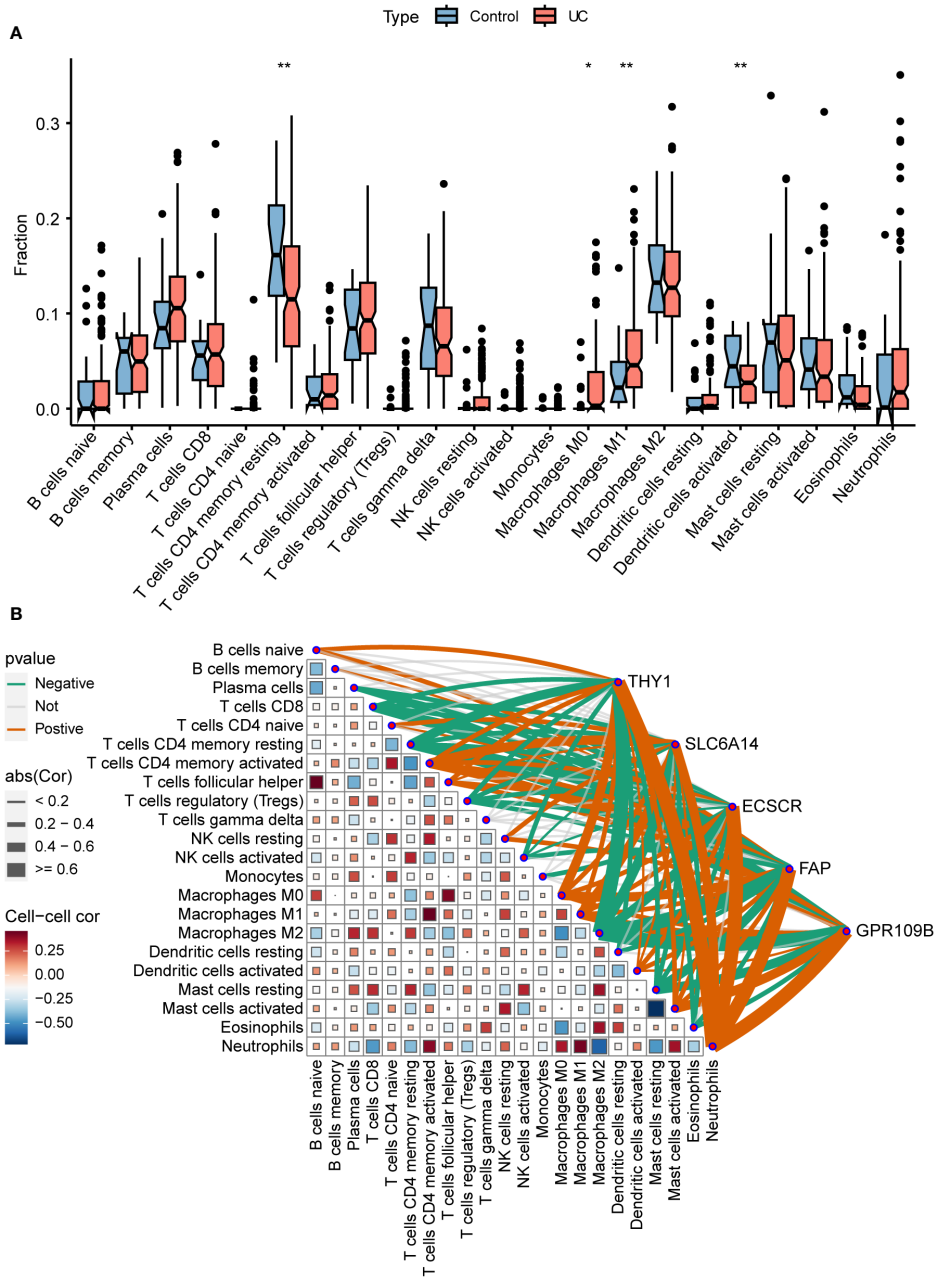
Immune infiltration and correlation. **(A)** Differences in infiltrating immune cells between normal control and UC. **(B)** Correlation of signature genes and 22 immune cell types. *P < 0.05, **P < 0.01.

To more precisely identify the colon immune cells associated with UC, the levels of 22 immune cell types were assessed in colon samples using CIBERSORT (**Figure 7A**). In comparison to healthy controls, UC patients exhibited elevated levels of M0 and M1 macrophages, along with decreased levels of resting CD4^+^ T memory cells and activated dendritic cells.

The association between immune cells and gene expression levels in the model was examined using Pearson correlation analysis (**Figure 7B**). The genes demonstrated a negative correlation with CD8^+^ T cells, resting memory CD4^+^ T cells, regulatory T cells, and M2 macrophages. Conversely, they exhibited a positive correlation with activated memory CD4^+^ T cells, M0 macrophages, M1 macrophages, activated mast cells, and neutrophils. This indicates that the varied expression of biomarkers has an impact on immune infiltration in UC.

### Signature gene expression is strongly associated with different types of UC and drug efficacy

**Figure 8:**
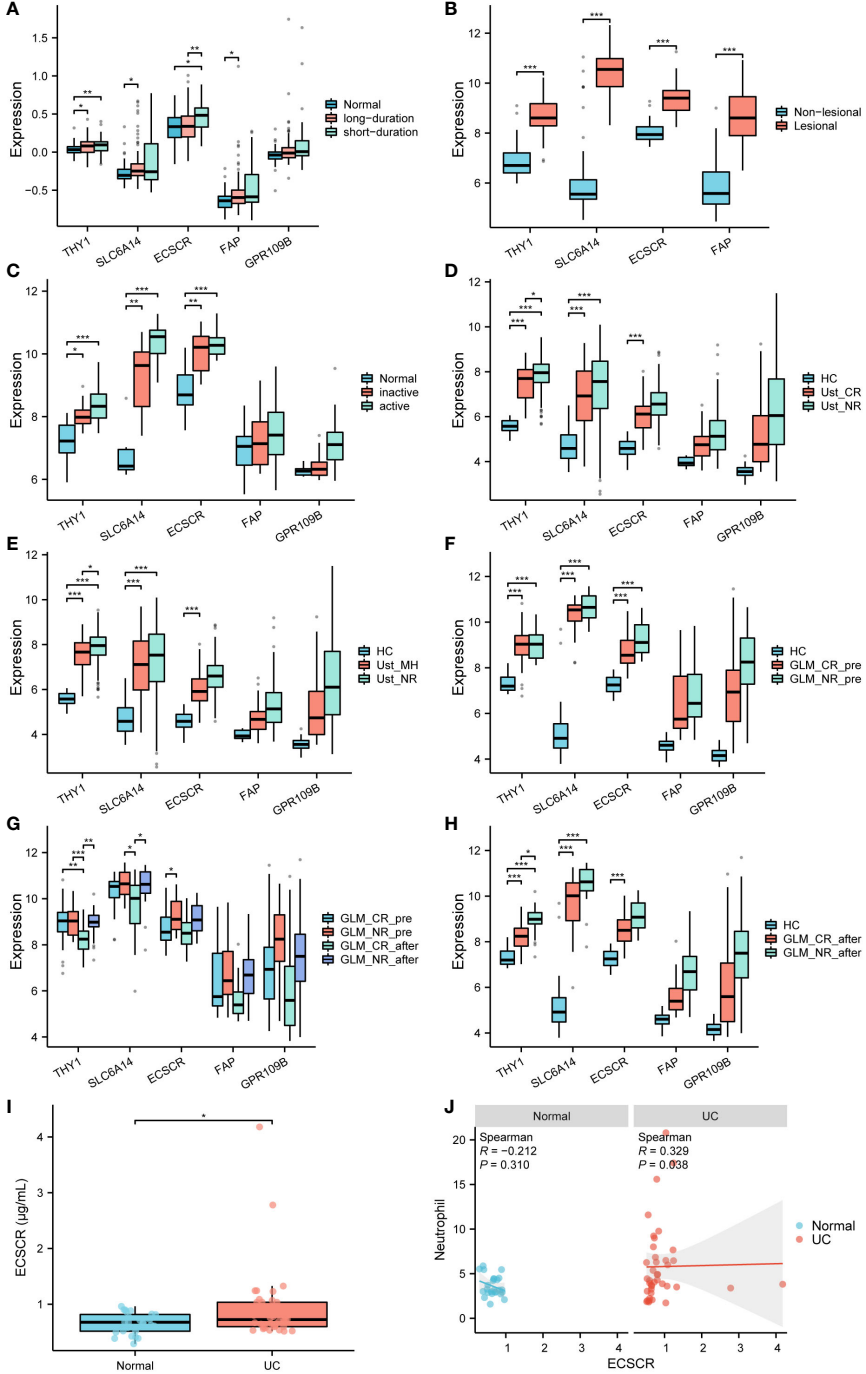
External validation of signature genes. **(A)** Differential expression of signature genes in normal control, long duration UC and short duration UC. **(B)** Differential expression of signature genes in lesional and non-lesional colonic tissues of UC patients. **(C)** Differential expression of signature genes in normal control, inactive and active UC. **(D, E)** Differential expression of signature genes in the colonic mucosal of HC (healthy control), Ust_CR (UC patients in clinical remission before ustekinumab therapy), Ust_NR (UC patients not responding before ustekinumab therapy), and Ust_MH (UC patients in mucosal healing before ustekinumab therapy). **(F–H)** Differential expression of signature genes in the colonic mucosa of healthy controls, UC patients in the non-response and response groups before and after GLM treatment. **(I)** Contents of ECSCR in serum of normal and UC patients were determined by ELISA. **(J)** Correlation between ECSCR and neutrophil levels. *P < 0.05, **P < 0.01, ***P < 0.001.

In the GSE11223 dataset, UC patients were stratified based on the duration of illness. Among the genes examined, namely THY1, SLC6A14, and FAP, significantly higher expression levels were observed in long-term UC (≥ 10 years). Conversely, in short-term UC (< 10 years), only THY1 exhibited elevated expression. Notably, ECSCR demonstrated increased expression in short-term UC but not in long-term UC (**Figure 8A**).

In the GSE107499 dataset, variations in the expression of colonic tissues were identified between inflamed and uninflamed states in UC (**Figure 8B**). The inflamed tissues exhibited a significant upregulation of signature genes compared to uninflamed tissues.

The GSE53306 dataset explores changes in gene expression among normal, active, and inactive colon tissues in patients with UC (**Figure 8C**). THY1, SLC6A14, and ECSCR exhibited a significant increase in both active and inactive UC tissues compared to normal tissue. However, there was no significant difference in their expression levels between active and inactive UC.

GSE206285 was to assess the expression profile of baseline biopsy samples from patients with moderate-to-severe UC treated with the IL-12/IL-23 inhibitor ustekinumab (Ust). THY1 levels were significantly lower in the responsive and mucosal healed UC patients compared to the non-responsive group before treatment with Ust. There was a slight decrease in SLC6A14 and ECSCR levels (**Figures 8D, E**).

The GSE92415 dataset comprises expression profiles from biopsy samples of UC patients treated with golimumab (GLM). Before the initiation of GLM treatment, patients with active UC exhibited higher expression levels of THY1, SLC6A14, and ECSCR compared to healthy controls. Following GLM treatment, although the expression of THY1 was reduced in the clinical remission group, the expression levels of THY1, SLC6A14, and ECSCR did not fully revert to those observed in healthy controls (**Figures 8F–H**).

The GSE92415 cohort contains information on the Mayo score for all samples. The results showed that according to the Mayo score > 5 was the high scoring group and the rest was the low scoring group, in the high scoring group the gene expression was significantly upregulated in the model. Correlation analysis further confirmed that gene expression in the model was positively correlated with Mayo score (**Supplementary Figure S1**).

ELISA of clinical serum samples showed that ECSCR was upregulated in the serum of UC patients and positively correlated with neutrophil levels (**Figures 8I, J**).

## Discussion

UC is characterized by a chronic, recurring IBD with a higher incidence rate (33). The low early diagnosis rate of UC, attributed to the absence of reliable biomarkers, leads to a delay in achieving initial remission of the disease (34). In order to enhance mucosal healing and remission rates, the identification of novel and effective diagnostic biomarkers accurately reflecting UC is imperative. This study employs RRA, WGCNA, and machine learning techniques to identify key signature genes. Validation of these signature genes is conducted using multiple patient datasets, encompassing disease onset and medication details, along with clinical samples. The outcomes of this research are anticipated to contribute significantly to the discovery of new biomarkers for the diagnosis and treatment of UC.

In this study, 212 intersecting genes were identified through RRA and WGCNA analyses. Subsequent GO analysis revealed that these DEGs were associated with leukocyte migration, humoral immune response, cytotaxis, and leukocyte chemotaxis, suggesting a pivotal role of leukocytes in the progression of UC. Neutrophils, crucial leukocyte cells, were observed in the epithelium, indicating histological UC activity and serving as an early core event in UC (35). These neutrophils coexisted with inflammation. The KEGG analysis demonstrated significant enrichment of genes related to the IL-17 signaling pathway, TNF signaling pathway, PI3K-Akt signaling pathway, RA and other immune-related pathways. Similarly, GSVA analysis indicated a significant activation of these pathways in UC patients, consistent with previous studies (7, 18). Intriguingly, these intersecting genes are not only associated with UC pathogenesis but also with RA. A large-sample meta-analysis suggested that individuals with inflammatory bowel inflammation are at a higher risk of developing RA (36, 37), indicating shared pathogenic factors for both UC and RA at the molecular level.

Five biomarkers (THY1, SLC6A14, ECSCR, FAP, and GPR109B) significantly associated with UC have been identified through machine learning analysis. A prediction model was developed, and ROC analysis demonstrated the model’s and its UC signature gene’s excellent discriminatory ability in distinguishing UC samples from healthy control samples. Furthermore, it is suggested that the UC signature gene may be closely linked to UC, providing a foundation for understanding the disease’s etiology and discovering potential new therapies. THY1 is highly expressed in synovial fibroblasts in RA, which can invade and degrade cartilage by secreting inflammatory cytokines and chemokines, while stimulating osteoclasts, leading to bone erosion (38). A correlation between RA and UC (OR 1.082; 95% CI 1.002–1.168; P = 0.044) was found by Mendelian randomization analysis (39). In addition, THY1, implicated in cell adhesion during inflammation, exhibits aberrant methylation associated with UC (40). These findings suggest that THY1 may be involved in the co-morbid processes of RA and UC. SLC6A14, also known as a Na^+^/Cl^−^ driven amino acid transporter B, is up-regulated in both rectal and caecal mucosa in UC patients (41). Studies indicate that SLC6A14 expression is upregulated in UC patients, potentially contributing to colonic inflammation by regulating glutamine and nitric oxide synthase 2 and may also contribute to UC via the C/EBPβ-PAK6 axis Iron death in epithelial cells (42–44). Moreover, there has been a suggestion that SLC6A14 contributes to the death of UC cells by controlling NLRP3 (45). FAP is a significant marker of cancer-associated fibroblasts and is closely linked to colorectal cancer invasion and metastasis. Additionally, the extent, duration, and severity of inflammation in UC are tied to a higher risk of colitis-associated colorectal cancer (CAC) (13). Furthermore, FAP levels are unusually high in UC. Further investigation is needed to determine if FAP plays a crucial role in the progression of UC to CAC. GPR109B, abundantly expressed in human neutrophils, plays a role in inflammatory processes, including UC. Targeting GPR109B in therapeutic strategies may prove beneficial in diseases characterized by inflammation, such as UC (46). Furthermore, the five aforementioned biomarkers are positively associated with UC activation pathways and negatively correlated with UC down-regulated signaling pathways, supporting their significance in UC development and progression.

Furthermore, notable variations in the composition of immune cells were observed between UC and control samples. UC patients exhibited a higher abundance of M1 macrophages compared to controls, while there was no difference in M2 macrophages. This observation aligns with the understanding that UC is primarily associated with the presence of pro-inflammatory M1 macrophages, but not anti-inflammatory M2 macrophages (47). Additionally, it was found that resting CD4^+^ T memory cells and activated dendritic cells were significantly lower in UC patients, contradicting previous findings (48). These differences may be attributed to the utilization of different datasets or the presence of unbalanced data in prior studies. Surprisingly, five biomarkers were found to have a significant positive correlation with macrophage M0 and M1 infiltration in the model. However, there is a scarcity of studies investigating the impact of these genes on immune cells in UC patients. Moreover, the genes in the model are positively correlated with neutrophils. It has been demonstrated that large numbers of neutrophils infiltrate the UC colonic mucosa, releasing serine proteases, matrix metalloproteinases, and myeloperoxidases through the production of reactive oxygen species, which directly cause tissue damage and produce typical crypt abscesses (13). Additionally, ELISA of clinical serum samples showed that ECSCR was upregulated in the serum of UC patients and positively correlated with neutrophil levels. These findings suggest that genes in the model are closely associated with neutrophil infiltration in UC. Our findings offer novel insights into the potential role of these genes in UC immunomodulation. Therefore, further investigation into the function of immune cells in the progression of UC is warranted.

Characterized gene expression has been confirmed to be strongly associated with various types of UC and the effectiveness of drugs in different datasets. Additionally, the diagnostic efficacy of ECSCR for UC has been further validated using clinical samples. Our hope is that these findings will present novel strategies for the diagnosis and treatment of UC. However, it is important to recognize certain limitations in this study. Firstly, the clinical data used were obtained from public databases, and the clinical information of the samples was incomplete, which hindered the exploration of the correlation between these characterized genes and clinical features. Secondly, no *in vivo* or *in vitro* experiments were conducted for validation. Therefore, further studies will be necessary to provide compelling evidence for our results.

In summary, novel targets such as THY1, SLC6A14, ECSCR, FAP, and GPR109B have been discovered in our findings, which could potentially play a role in the development of UC and serve as reliable diagnostic biomarkers for UC. Additionally, these new targets exhibit strong associations with various signaling pathways and immune cells involved in UC, thereby offering fresh insights into the underlying mechanisms of this condition.

## Data availability statement

The original contributions presented in the study are included in the article/**Supplementary Material**. Further inquiries can be directed to the corresponding authors.

## Ethics statement

The Ethics Committee of The First Affiliated Hospital of Soochow University reviewed and approved the study (ID:2024296).

## Author contributions

BF: Data curation, Investigation, Writing – original draft. YZ: Conceptualization, Formal analysis, Methodology, Writing – review & editing. LQ: Funding acquisition, Project administration, Resources, Writing – original draft. QT: Conceptualization, Resources, Visualization, Writing – original draft. ZZ: Methodology, Software, Writing – original draft. SZ: Resources, Validation, Visualization, Writing – original draft. JQ: Investigation, Software, Supervision, Writing – original draft. XZ: Formal analysis, Project administration, Resources, Writing – review & editing. CH: Conceptualization, Formal analysis, Validation, Writing – review & editing. YL: Funding acquisition, Validation, Writing – review & editing.
