# Supplementary material for: Evaluating the significance of ECSCR in the diagnosis of ulcerative colitis and drug efficacy assessment
Source: Front Immunol. 2024 Aug 7;15:1426875. doi: 10.3389/fimmu.2024.1426875 (PMC11335526; doi:10.3389/fimmu.2024.1426875)

Figure S1: Correlation between genes in the model and disease severity. (A) Gene expression in the model significantly increased in the high scoring group. (B) A positive association between gene expression in the model and Mayo score.

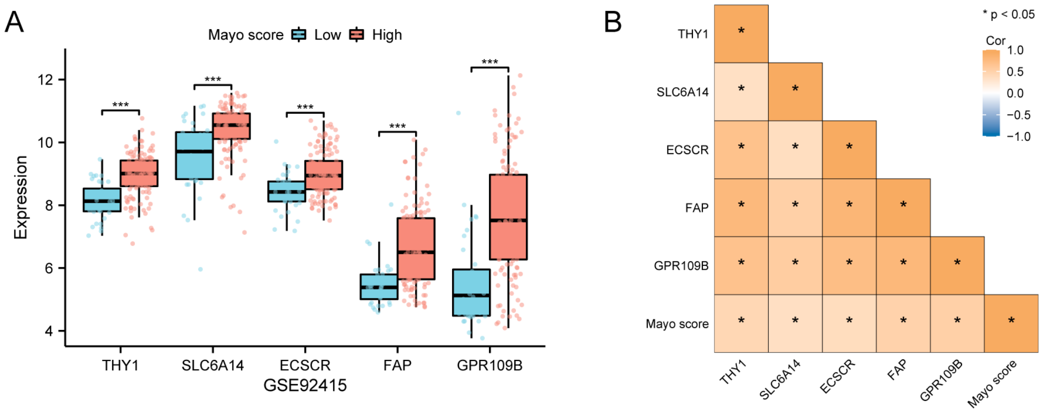

Supplement: Supplementary file 1 [file Image_1.pdf]
